# (*S*)-1-(2-Ammonio-3-methyl­butyl)-1,2-dihydro­pyridin-2-iminium dibromide

**DOI:** 10.1107/S1600536808012154

**Published:** 2008-05-10

**Authors:** Yifeng Wang, Jixv Zhang, Hui Chen, Shuping Luo

**Affiliations:** a State Key Laboratory Breeding Base of Green Chemistry–Synthesis Technology, Zhejiang University of Technology, Hangzhou 310014, People’s Republic of China

## Abstract

In the title compound, C_10_H_19_N_3_^2+^·2Br^−^, the plane of the three butyl C atoms nearest to the pyridine ring is almost perpendicular to the ring [dihedral angle = 84.80 (2)°]. The N atom of the ammonium group is displaced by 1.150 (8) Å from the plane of these three C atoms. The iminium N atom lies on the opposite side of this plane. The crystal structure is stabilized by hydrogen bonds between the N and Br atoms, as well as by inter­molecular C—H⋯Br inter­actions.

## Related literature

For the synthesis of (*S*)-1-bromo-3-methyl­butan-2-amine hydro­bromide, see: Xu *et al.* (2006 ▶). For related literature, see: Luo *et al.* (2006 ▶).
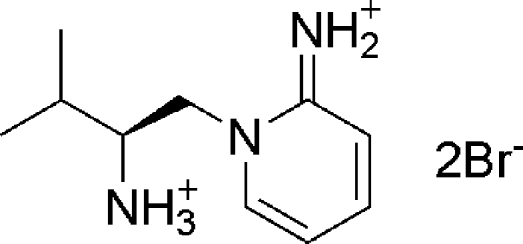


## Experimental

### 

#### Crystal data

C_10_H_19_N_3_^2+^·2Br^−^
*M* *_r_* = 341.10
Monoclinic, 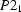

*a* = 5.9311 (11) Å
*b* = 12.456 (2) Å
*c* = 9.6807 (18) Å
β = 99.733 (3)°
*V* = 704.9 (2) Å^3^
*Z* = 2
Mo *K*α radiation
μ = 5.73 mm^−1^
*T* = 293 (2) K
0.45 × 0.34 × 0.20 mm

#### Data collection

Bruker SMART APEX CCD diffractometer
Absorption correction: multi-scan (*SADABS*; Sheldrick, 1996 ▶) *T*_min_ = 0.381, *T*_max_ = 1.000 (expected range = 0.119–0.313)
4117 measured reflections
2307 independent reflections
2054 reflections with *I* > 2σ(*I*)
*R*_int_ = 0.038

#### Refinement

*R*[*F* ^2^ > 2σ(*F* ^2^)] = 0.041
*wR*(*F* ^2^) = 0.092
*S* = 0.99
2307 reflections
147 parameters
3 restraints
H atoms treated by a mixture of independent and constrained refinement
Δρ_max_ = 0.91 e Å^−3^
Δρ_min_ = −0.71 e Å^−3^
Absolute structure: Flack (1983 ▶), 696 Friedel pairs
Flack parameter: 0.06 (2)

### 

Data collection: *SMART* (Bruker, 2001 ▶); cell refinement: *SAINT-Plus* (Bruker, 2000 ▶); data reduction: *SAINT-Plus*; program(s) used to solve structure: *SHELXS97* (Sheldrick, 2008 ▶); program(s) used to refine structure: *SHELXL97* (Sheldrick, 2008 ▶); molecular graphics: *SHELXTL* (Sheldrick, 2008 ▶); software used to prepare material for publication: *SHELXTL*.

## Supplementary Material

Crystal structure: contains datablocks global, I. DOI: 10.1107/S1600536808012154/cs2071sup1.cif

Structure factors: contains datablocks I. DOI: 10.1107/S1600536808012154/cs2071Isup2.hkl

Additional supplementary materials:  crystallographic information; 3D view; checkCIF report

## Figures and Tables

**Table 1:** Hydrogen-bond geometry (Å, °)

*D*—H⋯*A*	*D*—H	H⋯*A*	*D*⋯*A*	*D*—H⋯*A*
N2—H2*B*⋯Br2	0.84 (7)	2.55 (7)	3.368 (7)	167 (8)
N2—H2*A*⋯Br1^i^	0.84 (8)	2.53 (8)	3.357 (6)	168 (10)
N3—H3*C*⋯Br2^ii^	0.89	2.50	3.369 (5)	166
N3—H3*B*⋯Br1	0.89	2.46	3.238 (5)	147
N3—H3*A*⋯Br2^iii^	0.89	2.43	3.281 (5)	160
C3—H3⋯Br1^iv^	0.93	3.02	3.892 (8)	157
C4—H4⋯Br1^v^	0.93	2.91	3.748 (8)	150
C6—H6*A*⋯Br1^vi^	0.97	2.96	3.528 (7)	119
C5—H5⋯Br2^ii^	0.93	2.83	3.721 (7)	162
C8—H8⋯Br2	0.98	2.93	3.793 (7)	147

Symmetry codes: (i) 
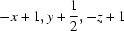
; (ii) 
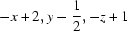
; (iii) 
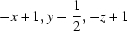
; (iv) 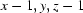; (v) 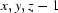; (vi) 
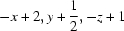
.

**Fig. 1.**
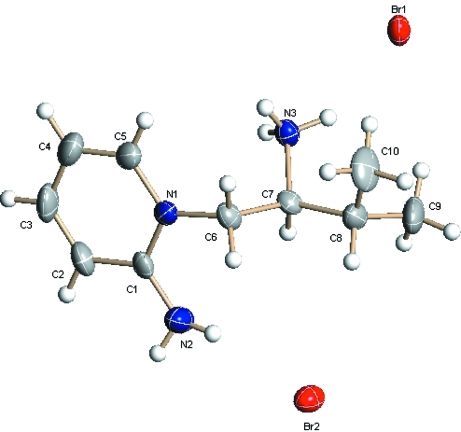
The asymmetric unit of the title compound with the atomic labeling scheme. Displacement ellipsoids are drawn at the 50% probability level.

**Fig. 2.**
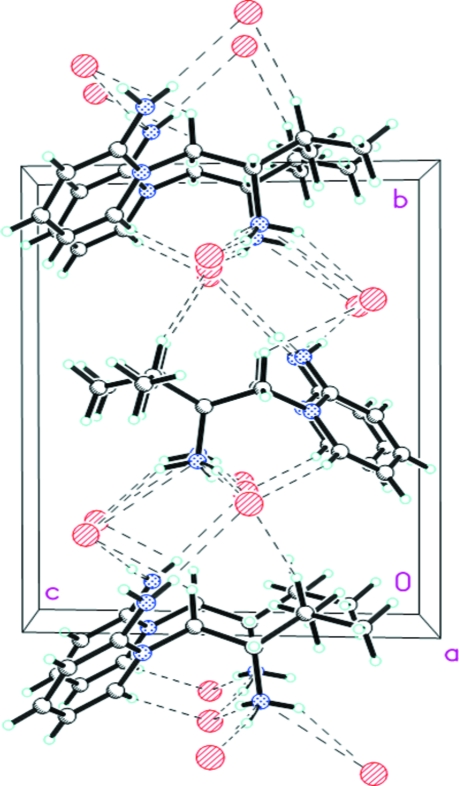
The molecular packing of the title compound showing H-bridge interactions between cationic-anionic groups.

## supplementary crystallographic information

### Comment

Ionic liquids, specially functional ionic liquids, have received growing attention recently due to their tuneable features for various chemical tasks. (S. Luo, *et al.*, 2006). The title compound, readily synthesized from commercially available *L*-valine and 2-aminopyridine, might have potential utilities in some specific chemical tasks, when it is converted into a kind of functional ionic liquid by neutralization with sodium hydroxide. The structure of (*S*)-1-(2-ammonio-3-methylbutyl)pyridin-2(1*H*)-iminium dibromide is shown in Fig. 1.

The crystal is built of doubly protonated cations and bromide anions. The protonation of the amines is appropriate like in the scheme, for the C1—N2 bond distance reveals its double bond property. The dihedral angle between the plane of three alkyl carbons C6/C7/C8 and the pyridine ring is 84.80 (2) °, which means the two planes are approximately perpendicular to one another. The atom N3 of the ammonium group bonded to the alkyl chain is displaced from the plane of three carbons C6/C7/C8 by 1.150 (8) Å. The iminium N2 lies on the opposite side of this plane. The crystal structure is stablized by hydrogen-bonds between the atoms N and Br as well as by intermolecular C—H—Br interactions. The molecular packing of the title compound showing H-bridge interactions between cationic-anionic groups is shown in Fig. 2.

### Experimental

The title compound was synthesized by treating 2-aminopyridine (0.94 g,10 mmol) with (*S*)-1-bromo-3-methylbutan-2-amine hydrobromide (2.47 g,10 mmol) in MeCN (30 ml) under stirring at 353 K for 24 h (yield 81%). The compound (*S*)-1-bromo-3-methylbutan-2-amine hydrobromide was obtained from commercially available *L*-valine by reduction with NaBH_4_ and subsequent bromination with PBr_3_ (Xu *et al.*, 2006). Suitable crystals of the title compound were obtained by slow evaporation of an ethanol solution at room temperature.

### Refinement

All carbon-bonded H atoms were placed in calculated positions with C—H = 0.93 Å (aromatic), C—H = 0.98 Å (*sp*), C—H = 0.93 Å (sp2), C—H = 0.96 Å(sp3) and refined using a riding model, with *U*_iso_(H)=1.2_eq_(C). N-bound H atoms were located in a difference map and refined with an N—H distance restraint of 0.86 (3) Å.

### Crystal data

**Table:**

C_10_H_19_N_3_^2+^·2Br^–^	*F*_000_ = 340
*M**_r_* = 341.10	*D*_x_ = 1.607 Mg m^−^^3^
Monoclinic, *P*2_1_	Mo *K*α radiation λ = 0.71073 Å
*a* = 5.9311 (11) Å	Cell parameters from 1818 reflections
*b* = 12.456 (2) Å	θ = 5.4–53.4º
*c* = 9.6807 (18) Å	µ = 5.73 mm^−^^1^
β = 99.733 (3)º	*T* = 293 (2) K
*V* = 704.9 (2) Å^3^	Prismatic, colorless
*Z* = 2	0.45 × 0.34 × 0.20 mm

### Data collection

**Table:**

Bruker SMART APEX CCD diffractometer	2307 independent reflections
Radiation source: fine-focus sealed tube	2054 reflections with *I* > 2σ(*I*)
Monochromator: graphite	*R*_int_ = 0.038
*T* = 293(2) K	θ_max_ = 27.0º
φ and ω scans	θ_min_ = 2.1º
Absorption correction: multi-scan(SADABS; Sheldrick, 1996)	*h* = −7→7
*T*_min_ = 0.381, *T*_max_ = 1.000	*k* = −12→15
4117 measured reflections	*l* = −12→12

### Refinement

**Table:**

Refinement on *F*^2^	Hydrogen site location: inferred from neighbouring sites
Least-squares matrix: full	H atoms treated by a mixture of independent and constrained refinement
*R*[*F*^2^ > 2σ(*F*^2^)] = 0.041	*w* = 1/[σ^2^(*F*_o_^2^) + (0.0533*P*)^2^] where *P* = (*F*_o_^2^ + 2*F*_c_^2^)/3
*wR*(*F*^2^) = 0.092	(Δ/σ)_max_ < 0.001
*S* = 0.99	Δρ_max_ = 0.91 e Å^−^^3^
2307 reflections	Δρ_min_ = −0.71 e Å^−^^3^
147 parameters	Extinction correction: none
3 restraints	Absolute structure: Flack (1983), 696 Friedel pairs
Primary atom site location: structure-invariant direct methods	Flack parameter: 0.06 (2)
Secondary atom site location: difference Fourier map	

### Fractional atomic coordinates and isotropic or equivalent isotropic displacement parameters (Å^2^)

**Table:**

	*x*	*y*	*z*	*U*_iso_*/*U*_eq_	
Br1	1.02019 (11)	0.21057 (4)	0.84434 (6)	0.03666 (18)	
Br2	0.61945 (10)	0.78903 (5)	0.54550 (7)	0.03742 (19)	
N1	0.7631 (8)	0.4733 (4)	0.2958 (5)	0.0269 (11)	
N2	0.4665 (11)	0.5923 (5)	0.3096 (7)	0.0398 (14)	
N3	0.9054 (9)	0.3672 (4)	0.5746 (5)	0.0291 (11)	
H3A	0.7799	0.3302	0.5406	0.044*	
H3B	0.9481	0.3518	0.6651	0.044*	
H3C	1.0172	0.3496	0.5280	0.044*	
C1	0.5506 (11)	0.5110 (5)	0.2454 (6)	0.0295 (14)	
C2	0.4223 (13)	0.4609 (6)	0.1291 (7)	0.0404 (17)	
H2	0.2766	0.4863	0.0937	0.048*	
C3	0.5050 (15)	0.3772 (7)	0.0677 (7)	0.052 (2)	
H3	0.4149	0.3427	−0.0072	0.062*	
C4	0.7331 (15)	0.3408 (7)	0.1176 (8)	0.052 (2)	
H4	0.7975	0.2856	0.0725	0.062*	
C5	0.8497 (13)	0.3880 (6)	0.2301 (7)	0.0378 (16)	
H5	0.9953	0.3628	0.2661	0.045*	
C6	0.9101 (11)	0.5224 (6)	0.4176 (7)	0.0314 (14)	
H6A	0.8922	0.5998	0.4119	0.038*	
H6B	1.0686	0.5060	0.4131	0.038*	
C7	0.8569 (10)	0.4843 (5)	0.5581 (6)	0.0274 (13)	
H7	0.6928	0.4945	0.5567	0.033*	
C8	0.9855 (11)	0.5495 (6)	0.6818 (7)	0.0369 (16)	
H8	0.9549	0.6256	0.6605	0.044*	
C9	0.8975 (15)	0.5251 (8)	0.8154 (7)	0.057 (2)	
H9A	0.9414	0.4535	0.8456	0.086*	
H9B	0.7338	0.5309	0.7994	0.086*	
H9C	0.9614	0.5753	0.8867	0.086*	
C10	1.2447 (12)	0.5333 (8)	0.6994 (8)	0.057 (2)	
H10A	1.3194	0.5787	0.7734	0.086*	
H10B	1.2962	0.5514	0.6135	0.086*	
H10C	1.2809	0.4596	0.7224	0.086*	
H2A	0.355 (12)	0.623 (8)	0.260 (9)	0.09 (4)*	
H2B	0.525 (13)	0.636 (6)	0.371 (7)	0.06 (3)*	

### Atomic displacement parameters (Å^2^)

**Table:**

	*U*^11^	*U*^22^	*U*^33^	*U*^12^	*U*^13^	*U*^23^
Br1	0.0508 (4)	0.0348 (4)	0.0231 (3)	−0.0033 (3)	0.0026 (3)	−0.0009 (3)
Br2	0.0309 (3)	0.0355 (4)	0.0448 (4)	0.0023 (3)	0.0033 (3)	−0.0065 (3)
N1	0.033 (3)	0.025 (3)	0.024 (3)	0.001 (2)	0.007 (2)	0.004 (2)
N2	0.040 (4)	0.035 (4)	0.041 (4)	0.006 (3)	−0.002 (3)	−0.003 (3)
N3	0.032 (3)	0.029 (3)	0.026 (3)	−0.004 (2)	0.004 (2)	0.003 (2)
C1	0.041 (4)	0.028 (4)	0.018 (3)	−0.001 (3)	0.002 (3)	0.006 (3)
C2	0.042 (4)	0.051 (5)	0.025 (3)	−0.004 (3)	−0.005 (3)	0.006 (3)
C3	0.074 (5)	0.054 (5)	0.025 (4)	−0.011 (4)	−0.002 (4)	−0.012 (4)
C4	0.077 (6)	0.049 (5)	0.032 (4)	0.006 (4)	0.014 (4)	−0.010 (3)
C5	0.051 (4)	0.038 (4)	0.026 (4)	0.009 (3)	0.012 (3)	0.003 (3)
C6	0.031 (3)	0.032 (4)	0.028 (3)	−0.003 (3)	−0.004 (3)	0.001 (3)
C7	0.021 (3)	0.030 (3)	0.029 (3)	0.001 (2)	−0.001 (2)	0.003 (3)
C8	0.046 (4)	0.030 (4)	0.031 (4)	0.002 (3)	−0.004 (3)	−0.006 (3)
C9	0.066 (5)	0.077 (6)	0.025 (4)	0.014 (5)	−0.003 (4)	−0.015 (4)
C10	0.035 (4)	0.088 (7)	0.043 (5)	−0.016 (4)	−0.008 (3)	−0.014 (5)

### Geometric parameters (Å, °)

**Table:**

Br1—H3B	2.4578	C4—C5	1.325 (10)
Br2—H2B	2.55 (7)	C4—H4	0.9300
N1—C1	1.356 (8)	C5—H5	0.9300
N1—C5	1.380 (8)	C6—C7	1.523 (9)
N1—C6	1.476 (8)	C6—H6A	0.9700
N2—C1	1.329 (9)	C6—H6B	0.9700
N2—H2A	0.84 (8)	C7—C8	1.538 (9)
N2—H2B	0.84 (7)	C7—H7	0.9800
N3—C7	1.490 (8)	C8—C9	1.506 (11)
N3—H3A	0.8900	C8—C10	1.531 (10)
N3—H3B	0.8900	C8—H8	0.9800
N3—H3C	0.8900	C9—H9A	0.9600
C1—C2	1.396 (9)	C9—H9B	0.9600
C2—C3	1.334 (11)	C9—H9C	0.9600
C2—H2	0.9300	C10—H10A	0.9600
C3—C4	1.431 (11)	C10—H10B	0.9600
C3—H3	0.9300	C10—H10C	0.9600
			
C1—N1—C5	119.8 (6)	C7—C6—H6A	108.8
C1—N1—C6	122.1 (5)	N1—C6—H6B	108.8
C5—N1—C6	118.2 (5)	C7—C6—H6B	108.8
C1—N2—H2A	114 (7)	H6A—C6—H6B	107.7
C1—N2—H2B	133 (6)	N3—C7—C6	109.6 (5)
H2A—N2—H2B	107 (9)	N3—C7—C8	111.9 (5)
C7—N3—H3A	109.5	C6—C7—C8	112.3 (6)
C7—N3—H3B	109.5	N3—C7—H7	107.6
H3A—N3—H3B	109.5	C6—C7—H7	107.6
C7—N3—H3C	109.5	C8—C7—H7	107.6
H3A—N3—H3C	109.5	C9—C8—C10	111.4 (6)
H3B—N3—H3C	109.5	C9—C8—C7	111.3 (6)
N2—C1—N1	119.7 (6)	C10—C8—C7	111.9 (6)
N2—C1—C2	121.4 (7)	C9—C8—H8	107.3
N1—C1—C2	118.8 (7)	C10—C8—H8	107.3
C3—C2—C1	121.2 (7)	C7—C8—H8	107.3
C3—C2—H2	119.4	C8—C9—H9A	109.5
C1—C2—H2	119.4	C8—C9—H9B	109.5
C2—C3—C4	119.7 (7)	H9A—C9—H9B	109.5
C2—C3—H3	120.2	C8—C9—H9C	109.5
C4—C3—H3	120.2	H9A—C9—H9C	109.5
C5—C4—C3	117.9 (7)	H9B—C9—H9C	109.5
C5—C4—H4	121.0	C8—C10—H10A	109.5
C3—C4—H4	121.0	C8—C10—H10B	109.5
C4—C5—N1	122.5 (7)	H10A—C10—H10B	109.5
C4—C5—H5	118.7	C8—C10—H10C	109.5
N1—C5—H5	118.7	H10A—C10—H10C	109.5
N1—C6—C7	113.7 (5)	H10B—C10—H10C	109.5
N1—C6—H6A	108.8		
			
C5—N1—C1—N2	179.3 (6)	C6—N1—C5—C4	−178.0 (7)
C6—N1—C1—N2	−2.4 (9)	C1—N1—C6—C7	82.3 (7)
C5—N1—C1—C2	1.2 (8)	C5—N1—C6—C7	−99.4 (7)
C6—N1—C1—C2	179.5 (6)	N1—C6—C7—N3	64.0 (7)
N2—C1—C2—C3	−177.9 (7)	N1—C6—C7—C8	−170.9 (5)
N1—C1—C2—C3	0.2 (10)	N3—C7—C8—C9	−67.1 (7)
C1—C2—C3—C4	−2.8 (11)	C6—C7—C8—C9	169.2 (6)
C2—C3—C4—C5	4.2 (11)	N3—C7—C8—C10	58.3 (8)
C3—C4—C5—N1	−3.0 (11)	C6—C7—C8—C10	−65.5 (8)
C1—N1—C5—C4	0.3 (10)		

### Hydrogen-bond geometry (Å, °)

**Table:**

*D*—H···*A*	*D*—H	H···*A*	*D*···*A*	*D*—H···*A*
N2—H2B···Br2	0.84 (7)	2.55 (7)	3.368 (7)	167 (8)
N2—H2A···Br1^i^	0.84 (8)	2.53 (8)	3.357 (6)	168 (10)
N3—H3C···Br2^ii^	0.89	2.50	3.369 (5)	166
N3—H3B···Br1	0.89	2.46	3.238 (5)	147
N3—H3A···Br2^iii^	0.89	2.43	3.281 (5)	160
C3—H3···Br1^iv^	0.93	3.02	3.892 (8)	157
C4—H4···Br1^v^	0.93	2.91	3.748 (8)	150
C6—H6A···Br1^vi^	0.97	2.96	3.528 (7)	119
C5—H5···Br2^ii^	0.93	2.83	3.721 (7)	162
C8—H8···Br2	0.98	2.93	3.793 (7)	147

Symmetry codes: (i) −*x*+1, *y*+1/2, −*z*+1; (ii) −*x*+2, *y*−1/2, −*z*+1; (iii) −*x*+1, *y*−1/2, −*z*+1; (iv) *x*−1, *y*, *z*−1; (v) *x*, *y*, *z*−1; (vi) −*x*+2, *y*+1/2, −*z*+1.

## References

[bb1] Bruker (2000). *SAINT-Plus* Bruker AXS Inc., Madison, Wisconsin, USA.

[bb2] Bruker (2001). *SMART* Bruker AXS Inc., Madison, Wisconsin, USA.

[bb3] Flack, H. D. (1983). *Acta Cryst.* A**39**, 876–881.

[bb4] Luo, S., Mi, X., Zhang, L., Liu, S., Xu, H. & Cheng, J. (2006). *Angew. Chem. Int. Ed.* **45**, 3093–3097. 10.1002/anie.200600048 16586518

[bb5] Sheldrick, G. M. (1996). *SADABS* University of Göttingen, Germany.

[bb6] Sheldrick, G. M. (2008). *Acta Cryst.* A**64**, 112–122. 10.1107/S0108767307043930 18156677

[bb7] Xu, D. Q., Luo, S. P., Yue, H. D., Wang, L. P., Liu, Y. K. & Xu, Z. Y. (2006). *Synlett*, **16**, 2569–2572.

